# Supplementary material for: Predation and fragmentation portrayed in the statistical structure of prey time series
Source: BMC Ecol. 2009 May 6;9:10. doi: 10.1186/1472-6785-9-10 (PMC2689204; doi:10.1186/1472-6785-9-10)
Supplement: Additional file 2 — Voles and related classes ODDox Documentation. ODDox documentation of the agent-based model (ALMaSS) applied by Hendrichsen et al. The documentation is started by activating main.html. [file 1472-6785-9-10-S2.zip › Vole_ODDox/page1.html]

ALMaSS ODDox: Crop Classes

- Main Page
- Related Pages
- Classes
- Files

# Crop Classes

Crop class documentation following ODDox protocol   
  

## Overview

## 1. Purpose

The Crop model simulates the farm operations, their timing, probability of occuring and inter-relationships with other farm operations on a single farmers field in the 'life' of a single crop of winter wheat. The aim is to generate a realistic sequence of farming operations that is available as input to all other parts of the ALMaSS system.

## 2. State variables and scales

The scale is a single cultivated ALMaSS habitat element. The element is considered homogenous w.r.t. farm operations (i.e. if some part of it does not have certain operations e.g. unsprayed margins, then this exists as a separate element). State variables consist of 'flags' denoting whether operations have been carried out e.g. for WinterWheat WinterWheat.h::WW\_AUTUMN\_PLOUGH, and a stack of events to be carried out. These events are listed in the enumerator e.g. WinterWheatToDo. The order of events, and which are carried out is determined by the code structure in the Do method e.g. WinterWheat::Do

## 3. Process Overview and Scheduling

Scheduling is determined by the calls to the Do method ( e.g. WinterWheat::Do ), which will be called each timestep (defined by the landscape as 1 day) as long as there are events to process. If an event is handled by Do then it is passed into a switch statement and a test taken to determine whether it should be carried out. In every case the result will either be that the event is queued up for the next day (unsuccessful) or that another event or events will be queued up for some time in the future.

---

## Design

## 4. Design Concepts

The Crop model is not an ABM/IBM model, but a management construct capable of making decisions about farming events based on its present state and inputs, hence the IBM concepts do not apply:  
Emergence - no higher order behaviour emerges from the winter wheat class.  
Adaptation - there is no adaptation.  
Fitness - there is no measure of fitness.  
Prediction - no predication is done by this class.  
Sensing - the winter wheat class does not sense its environment. However the vegetation growth model that each instance of the class is related to does.  
Stochasticity - this is built in in terms of probabilistic decisions taken by the farmer. These decisions are relegated to probabilities in the abscence of applicable rules. The probabilties are designed to create distributions of activities matching observed means.  
Collectives - there are no collectives.  
Observation - observation is in terms of the order and timing of events placed on the event queue.  
  

## 5. Initialisation

Intialisation requires only that the management plan is started. At this point the end time for the management plan is set and any adjustments due to any possible early starting crops are made by shortening the last farm operations to fit the starting point of the next crop.

## 6. Inputs

Inputs are the proportions of farmers carrying out different operations e.g. spraying. The majority of these are hard-coded since the management plan is based on data collected at a particular time. These figures do change with time hence a range of winter wheat managements might be needed to compare temporal scenarios. Dynamic inputs to the model are the result of the farmer attempting to carry out farming operations e.g. Farm::AutumnPlough. These will depend on those factors important for the particular operation and are under the control of the farm manager. Typical variables important here are weather and soil based data.

## 7. Interconnections

There are no submodels, but the crop management plan is strongly linked to the Farm and farming operations therein. Each instance of the Crop class will also be linked to an instance of a LandscapeElement which will model vegetation growth (crop and weed) and generalised insect biomass.  
  
The following is a list of descendent classes of Crop:   
Carrots   
CloverGrassGrazed1   
CloverGrassGrazed2   
FieldPeas   
Fodderbeet   
Maize   
OBarleyPeaCloverGrass   
OSBarleySilage   
OCarrots   
OCloverGrassGrazed1   
OCloverGrassGrazed2   
OCloverGrassSilage1   
OFieldPeas   
OFieldPeasSilage   
OFirstYearDanger   
OGrazingPigs   
Oats   
OOats   
OPermanentGrassGrazed   
OPotatoes   
OSpringBarley   
OSpringBarleyPigs   
OWinterBarley   
OWinterRape   
OWinterRye   
OWinterWheatUndersown   
PermanentSetAside   
PermanentGrassGrazed   
PermanentGrassLowGrazed   
Potatoes   
SpringBarleyCloverGrass   
SeedGrass1   
SeedGrass2   
SetAside   
SpringBarley   
SpringBarleySeed   
SpringBarleySilage   
Triticale   
WinterBarley   
WinterRape   
WinterRye   
WinterWheat   
WWheatPControl   
WWheatPToxicControl   
WWheatPTreatment   
AgroChemIndustryCereal   
WinterWheatStrigling   
WinterWheatStriglingSingle   
WinterWheatStriglingCulm   
SpringBarleyCloverGrassStrigling   
SpringBarleyStrigling   
SpringBarleyStriglingSingle   
SpringBarleyStriglingCulm   
MaizeStrigling   
WinterRapeStrigling   
WinterRyeStrigling   
WinterBarleyStrigling   
FieldPeasStrigling   
SpringBarleyPeaCloverGrassStrigling   
YoungForestCrop

---

Generated on Thu Jan 22 14:13:45 2009 for ALMaSS ODDox by 
 1.5.6
